# Differential Colonization Dynamics of Marine Biofilm-Forming Eukaryotic Microbes on Different Protective Coating Materials

**DOI:** 10.3390/polym11010161

**Published:** 2019-01-17

**Authors:** Yanhe Lang, Yuan Sun, Miao Yu, Yubin Ji, Lei Wang, Zhizhou Zhang

**Affiliations:** 1 Key Laboratory of Saline-alkali Vegetation Ecology Restoration in Oil Field (SAVER), Ministry of Education, Alkali Soil Natural Environmental Science Center (ASNESC), Northeast Forestry University, Harbin 150040, China; langyanhe@163.com; 2 Center of Marine Antifouling Engineering Technology of Shandong Province, School of Marine Science and Technology, Harbin Institute of Technology at Weihai, Weihai 264209, China; 3 School of Science, Harbin University of Commerce, Harbin 150076, China; yumiao2015@163.com (M.Y.); yunbinji@sina.com (Y.J.); 4 School of Chemistry and Chemical Engineering, Harbin Institute of Technology, Harbin 150001, China

**Keywords:** early-adherent eukaryotes, marine biofouling, natural biofilms, protective coatings, single-strand conformation polymorphism, surface properties

## Abstract

In this study, the actual anti-biofouling (AF) efficacy of three protective coatings, including a chlorinated rubber-based coating (C_0_) and two polydimethylsiloxane (PDMS)-based coatings (P_0_ and P_F_), were estimated via the static field exposure assays. The surface properties of these protective coatings, including surface wettability and morphology features, were characterized using the static water contact angle (WCA) and scanning electron microscope (SEM). The colonization and succession dynamics of the early-adherent biofilm-forming eukaryotic microbial communities occupied on these protective coatings were explored using the Single-stranded Conformation Polymorphism (SSCP) technique. The field data clearly revealed that coating P_0_ and P_F_ performed better in the long-term static submergence, as compared with the C_0_ surface, while coating P_F_ showed excellent AF efficacy in the field. Fingerprinting analysis suggested that the diversity, abundance, the clustering patterns, and colonization dynamics of the early-colonized eukaryotic microbes were significantly perturbed by these protective coatings, particularly by the P_F_ surfaces. These differential AF efficacy and perturbation effects would be largely ascribed to the differences in the wettability and surface nanostructures between the C_0_, P_0_ and P_F_ surfaces, as evidenced by WCA and SEM analysis.

## 1. Introduction

Undesirable biological colonization on the submerged synthetic materials in marine water is collectively termed as marine biofouling [1]. Marine biofouling can trigger negative impacts on a broad range of maritime activities, inducing remarkable economic loss for shipping industries, offshore oil platforms as well as industrial equipment [2]. Thus, biofouling mitigation is extremely significant for these marine-associated industries. Various novel antifouling (AF) materials capable of preventing biofouling have been highly explored in the past few decades [3].

Chlorinated rubber-based coatings, produced mainly by reacting natural rubber with chlorine, have wide commercial applications for the corrosion protection of artificial surfaces underwater, including carbon steels, marine buildings, and constructions, owing to their decent chemical properties and water resistance [4]. Iron oxides with excellent corrosion resistance are considered one of the most commonly used pigments in the chlorinated rubber resin [5], since the wide distribution of the coarse and fine particles in the paint films could contribute considerably to the reinforcement of the barrier action against the penetration of the natural seawater [6]. In addition, fouling-release (FR) materials, typically polydimethylsiloxane (PDMS)-based coatings, are preferred due to their fascinating characteristics, such as biocompatibility, elastomeric, optically transparent, amenable to fabrication and non-toxic releases [7], which have expanded maritime applications in recent years [8]. The significance of the optical transparency of the substratum has been emphasized by previous publications, and the substratum color is believed to play key roles in the formation of micro and macro fouling communities in the natural biofilms. Previously, both Dobretsov et al. [9] and Swain et al. [10] have confirmed that substratum color should be taken into account when undertaking short-term testing of AF coatings in future studies. As a result, the optical transparency would be helpful to minimize the impact of substratum color on the colonization dynamics of surface-associated pioneer fouling organisms.

Considering the wide applications of PDMS, inorganic nanoparticles with unique properties (e.g., carbon nanotubes, CNTs), were introduced into the PDMS matrix to impart the pure PDMS with more functionalities in the field of AF research [11,12]. Most inorganic nanoparticles with a high surface area to volume ratio and photo catalytic properties as reinforcing agents in a wide range of polymeric matrices were primarily responsible for their improved AF and FR properties. CNTs were excellent candidates as nanofillers, due to their superior properties including unique molecular structure, exceptional electrical, mechanical, thermal, antimicrobial properties, as well as their biodegradability through the global biogeochemical cycle [13]. However, although the photocatalytic reactions of carbon nanotubes have broad applications in the field of industrial catalysis, the photocatalytic properties contribute less to the improvement of anti-biofouling efficacy of polymeric matrices, such as PDMS, as Yang et al. reported in their earlier studies [14]. Meanwhile, nanoparticle incorporation would be contributable to the enhanced interactions between the AF coating surfaces and marine microorganisms [15]. Moreover, the gradually decreased ion release would also minimize the detrimental impacts on the non-target marine organisms as well as the marine environment. 

In the marine benthic environment, the submerged coating surfaces are often quickly colonized by early natural biofilms [16]. The natural biofilms have already attracted worldwide attention recently, since their presence can alter the surface properties of these substrata, and further modulate the initial recruitment and colonization process of macrofoulers [17], such as invertebrate larvae, algae spores, or other planktonic forms [18,19,20]. These artificial surfaces can also exert differential influences on the assemblages of either marine bacteria or eukaryotes in the natural biofilms [21]. Previously, many 16S rRNA gene surveys have evidenced the extensive presence of pioneer biofilm-forming prokaryotic microbes grown on different substrata [22,23,24,25]. However, there are no equivalent studies dedicated to the early eukaryotes microbes because of the lack of molecular markers for further inspection of the pioneer biofilm-forming eukaryotic microbes [26], although previous studies have revealed these pioneer microbial eukaryotes are key players in the marine ecosystems and fulfill crucial biological functions in the developmental process of pioneer natural biofilms formation on different substrata [27,28]. Therefore, understanding the dynamics of diversity, abundance and distributional patterns of pioneer microbial eukaryotes is critical to understanding the complex interactions between different protective coating surfaces and pioneer eukaryotic microbes, which can be further estimated by culture-independent PCR-based molecular fingerprinting methods, such as the Single-stranded Conformation Polymorphism (SSCP). This culture-independent technique has been broadly adopted to estimate the diversity of mixed microbial communities under different environmental conditions, although the SSCP patterns are sometimes too complex to be deciphered. 

Herein, in the current study, three protective coatings, including a chlorinated rubber-based anticorrosion paint (coating C_0_), the pristine PDMS (coating P_0_), and carboxyl-modified multi-walled CNTs (cMWCNTs)-filled PDMS composites (coating P_F_), were deployed in the western coast of Weihai, China, and examined under actual marine conditions. The impacts of these protective coatings on the initial colonization and successional dynamics of pioneer eukaryotic biofilm-forming communities were investigated during a two-week in situ deployment. Coating characterizations were carried out in order to screen the discrepancies of surface properties among different selected protective coatings. 

## 2. Materials and Methods 

### 2.1. Coating Formulation 

Carbon steel substrates (measuring 100 mm ×100 mm × 3 mm) were polished using abrasive paper of different grits. Afterwards, these steel panels were repeatedly washed with double distilled water (ddH_2_O), thoroughly rinsed with 70% (*v*/*v*) ethanol, and then air dried at room temperature. A commercially available chlorinated rubber-based iron oxide red paint (coating, C_0_) which was commonly used to protect the steel surfaces from corrosion, was kindly supplied by the Jiamei Company (Weihai, China). These pretreated steel panels were firstly coated by coating C_0_ prior to use, and then cured at room temperature for three days.

The silicone elastomer applied in the present study, i.e., pristine PDMS (P_0_) Sylgard 184 kit (Dow Corning, Midland, MI, USA), primarily consisted of two components, A and B, which required to be formulated in a ratio of 10:1 following the manufacturer’s specifications. The carboxyl-modified multi-walled CNTs (cMWCNTs, outer diameter, >50 nm; length, ~20 μm, surface area, >40 m^2^/g) applied in the current study was purchased from TimesNano company (Chengdu, China).

The cMWCNTs/PDMS nanocomposites (P_F_) were formulated according to the procedures described by Beigbeder et al. in their previous study [29]. Briefly, the cMWCNT filler has the priority to mechanically blend with Sylgard 184-part A at 1000 rpm for about half an hour at room temperature. Afterwards, the Sylgard 184-part B was well blended with the aforementioned PDMS mixtures, then stirred up at 500 rpm for 1 min. The final amount of cMWCNT in the PDMS matrix was adjusted to 0.1 % (*w*/*w*) of the dry weight of the composite. Then these PDMS-based mixtures (i.e., P_0_ and P_F_) were painted on the surfaces of the pretreated panels using a bar-coater, respectively, which can be cured at 6 h at 105 °C in an oven. The pretreated panels coated with coating C_0_ served as coating controls. A minimum of three specimens of each protective coatings were formulated for the subsequent statistical evaluation.

### 2.2. Static Ocean Exposure Assays

Marine field assays were conducted at a marina on the west coast of Weihai, China (Lat N 37°31′51′’; Long E 121°58′19′’), following the Chinese national standard (GB 5370-2007), i.e., a method for testing antifouling panels in shallow ocean submergence. All coating samples were immersed in deionized water for one week prior to submergence in order to minimize the possibility of any pollutants leaching from the production process once the field assays started. The coating samples applied to the tested panels were lowered into seawater from a static experimental wooden bridge within the distance between the adjacent coatings approximately 5 cm. These coating casts were horizontally suspended at a 1.5 m below the lowest tide level at different points in time (Layout see Supplementary Figure S1). Immersion under static conditions at 1.5 m was carried out at two stages, i.e., Stage I, from October 19, 2013 to March 18, 2014; Stage II, from March 25, 2014 to June 13, 2014, respectively. Three replicate panels were periodically retrieved at different exposure times in order to estimate the global biofouling conditions of each coating surface. 

### 2.3. Sampling the Natural Biofilms

A two-week marine in situ experiment was performed from April 11–25, 2014 under static conditions in order to obtain continuous biofilm samples taken from the C_0_, P_0_ and P_F_ surfaces for further fingerprinting analysis. Panels for sampling were formulated in triplicates for every protective coating surface throughout, which were randomly deployed at 1.5 m following similar immersion conditions. All retrieved panel replicates were thoroughly rinsed with distilled water to remove the temporarily adhered debris and attached epifauna/epiphytes. About a 8 cm × 8 cm area of each panel was meticulously sampled and totally scraped with sterile brushes. Afterwards, the triplicated biofilm samples collected from the same material surface and sampling date were put into one sterile Eppendorf tube together as a representative of all fouling replicates, and then they were maintained at −80 °C for the subsequent analysis.

### 2.4. Single-Stranded Conformation Polymorphism

The genomic DNA was extracted from the biofilm samples as Briand et al. previously described [30]. The Internal Transcribed Spacer-2 (ITS-2) region was amplified from the extracted genomic DNA of all fouling samples using the ITS3/ ITS4 primer pair: i.e., ITS3 (5’-GCA TCG ATG AAG AAC GCA GC-3’) and ITS4 (5’-TCC TCC GCT TAT TGA TAT GC-3’) as reported earlier [31]. The differential amplified PCR products of ITS-2 region were subjected to the detection of PCR-SSCP systems in order to characterize the early-adherent biofilm-forming eukaryotic microbes established in our previous studies [26]. In order to avoid the high rate of re-annealing of DNA strands after the initial denaturation leading to the formation of heteroduplexes, the asymmetric PCR reaction was performed using ITS4 only. For each set of PCR reactions, a negative control was included. The SSCP gel was visualized using silver nitrate staining for 10 min, and then placed in freshly made developing solution for 5 min, which contained 0.2% Sodium carbonate with the addition of formaldehyde up to 0.05%, and then terminated in 5% acetic acid for another 5 min. Afterwards, the eukaryotic SSCP patterns were recorded for further fingerprinting analysis. 

### 2.5. Coating Characterization

#### 2.5.1. Water Contact Angle Measurements

The static WCA measurement was conducted on a JGW-360A Contact Angle analyzer at room temperature by depositing a water drop on each coating surface based on the sessile drop technique. In brief, when the water contacted the surface of the substrate, the syringe was moved up, leaving the droplet on the surface without motion, thus obtaining static contact angle values [32]. These tested surfaces were carefully cleaned in deionized water prior to testing. Five different points on each coating surface were tested in order to obtain an average value. 

#### 2.5.2. Scanning Electron Microscopy Analysis

The surface nanostructures of coating C_0_, P_0_ and P_F_ were observed on a Hitachi S-4800 instrument SEM Scanning Electron Microscope (SEM, Hitachi Limited, Japan), which was operated at the acceleration voltage of 15.0 kV. It was noteworthy that all of the coating samples were sputter-coated with gold prior to characterization in order to minimize sample charging.

### 2.6. Data Analysis

The lanes and bands in different eukaryotic SSCP profiles were detected using the Quantity One software 4.6.2 (Bio-Rad, Hercules, CA, USA). The binary data matrices can be obtained based on the presence/absence of nucleic acid bands. The ecological indices, including Shannon diversity index, Simpson index, Abundance and Evenness index, were calculated using the Biodap software on the basis of the SSCP presence/absence matrix. The early-eukaryotic-diversity was primarily measured by the Shannon index and the Simpson index, whereas the distribution patterns of early eukaryotic microbial communities were measured by the Evenness index. One-way analysis of variance (ANOVA) was used to compare the statistical differences of the diversity, abundance, and distribution patterns of the early-adherent biofilm-forming eukaryotic microbial communities formed on different protective coating surfaces, using the GraphPad Prism 6.03 software (GraphPad Software, San Jose, CA, USA). The statistical significance was accepted at a P-value < 0.05 or P-value < 0.01. The phylogenetic tree of the early eukaryotic communities developed on the different protective coating surfaces at different points in time were constructed based on the Unweighted Pair-Group Method with Arithmetic means (UPGMA) using the Quantity One software 4.6.2 (Bio-Rad). The clustering patterns of the eukaryotic microbial communities were explored on the basis of the multidimensional scale (MDS) method using the SPSS19.0 software (IBM, Armonk, NY, USA).

## 3. Results and Discussion

### 3.1. Coating Characterization 

#### 3.1.1. Static WCA Measurements

Static water Contact angle (WCA) measurement is the primary data that revealed the degree of wettability on solid- liquid interfaces. According to the WCA measurements, the static θw value of three representative protective coatings (i.e., C_0_, P_0_ and P_F_ surfaces) ranged from 68.6 ± 1.5° and 114.3 ± 1.2°, respectively. Coating C_0_ were considered as hydrophilic surface, with the θw value (68.6 ± 1.5°) significantly lower than 90°. In contrast, the pristine PDMS (coating P_0_) showed a WCA of 96.8 ± 1.3°, which was higher than 90°, thus consequently considered as hydrophobic surface [23]. Furthermore, coating P_F_ (118.3 ± 1.6°) were significantly higher than 90°, which was more hydrophobic than the pristine PDMS. This indicated that the cMWCNTs incorporated in PDMS would result in significant changes in surface wettability. There were significant differences (P < 0.05) in the θw value between all these aforementioned protective coatings.

#### 3.1.2. SEM Characterization

**Figure 1:**
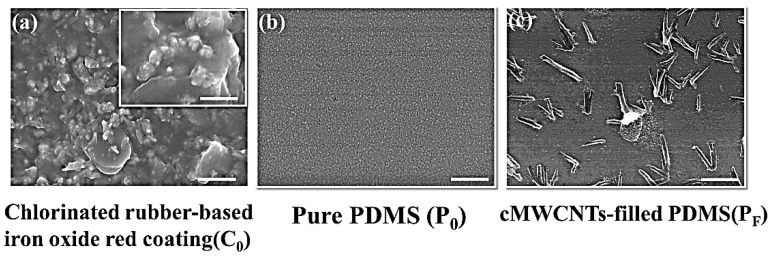
The scanning electron microscope (SEM) images of the three coatings (i.e., C_0_, P_0_ and P_F_ surfaces.) before immersion for comparison. The scale bars are 2 μm in main figures, and 200 nm in inset.

Figure 1 showed the SEM image of the morphological characteristics of three protective coating materials, which gave a visual depiction on the polymer surface morphology and aggregation of cMWCNTs in the PDMS matrix. Coating C_0_ and P_F_ possessed differential surface morphological features, which showed increased surface roughness in comparison with that of the plain PDMS (P_0_). In Figure 1a, it was clear that a number of nanoparticles with the size of 10 nm were found to be evenly distributed on the surface of chlorinated rubber-based coating (coating C_0_) and displayed clear surface structural patterns, which may greatly contribute to the enhanced roughness of the substrate. It is noticeable that the surface morphology coating C_0_ seemed rougher than that of the pristine PDMS (P_0_) and cMWCNTs/PDMS composites (coating P_F_), and displayed θw value (68.6 ± 1.5°) lower than 90°, thus resulting in the decreased hydrophilicity character. By contrast, based on the SEM image shown in Figure 1b,c, the surfaces of the P_0_ and P_F_ surfaces remained relatively smooth, only a few cMWCNT nanoparticles were well dispersed on the PF surfaces, which may be conducive to the increased roughness, since the θw value for coating P_F_ (118.3 ± 1.6°) was higher than 90°, therefore the increased coating roughness would have a positive effect on the higher θw value and further result in the increased hydrophobicity of the PDMS resin, which also correlated well with the Wenzel model reported in their earlier studies [33].

### 3.2. Field Exposure Studies

**Figure 2:**
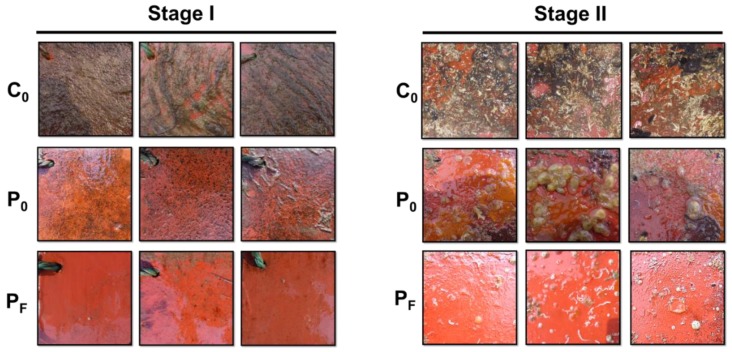
Images of the tested steel substrates coated with different coatings (i.e., C_0_, P_0_ and P_F_ surfaces) immersed in the natural seawater at different exposure times. Stage I: Oct.19, 2013-Mar.18, 2014, 150 days; Stage II: Mar.25, 2014-July.13, 2014,110 days.

Figure 2 displayed the field data obtained from the static ocean immersion assays. According to visual inspections, in stage I, the average temperature of the seawater being 9 °C, the panels coated with C_0_ were heavily covered with marine sediments, seaweeds, algae spores and microbial slime. However, the panels coated with the PDMS-based coatings (i.e., coating P_0_ and P_F_) exhibited less fouling than the C_0_ surface. Coating P_F_ proved to be efficient in biofouling mitigation with low colonization of the early colonizers. In contrast, in stage II, the average temperature of the seawater being 15°C, both coating C_0_ and P_0_ had fouled heavily and displayed wide coverage with macrofoulers. Specifically, coating C_0_ was mostly covered with both hard-foulings (juvenile mussels) and soft-foulings (Calcarina), plus marine sediments and slime, while coating P_0_ was terribly fouled mainly by some soft-foulings, mostly the sea squirt and a few tubeworms. However, unlike coating C_0_ and P_0_, coating P_F_ still showed excellent AF performance, with less tubeworms and adult barnacle colonization. 

On the basis of the field data, coating C_0_ did not perform well at the two exposure stages, which cannot resist the colonization of major fouling organisms, while the PDMS-based composites demonstrated high resistantance against the colonization of the early colonizers. Only coating P_F_ performed exceptionally well either in stage I or stage II, thereby being promising in future marine AF applications. It was noteworthy that the maritime application data on the AF performance of coating P_F_ remained insufficient. It requires static immersion assays conducted at different immersion sites and seasons to validate their effectiveness on travelling ships under actual marine environment condidtions, which will occur in our future studies. 

### 3.3. Analysis of the Pioneer Eukaryotic Biofilm Communities

#### 3.3.1. The Eukaryotic SSCP Fingerprints

**Figure 3:**
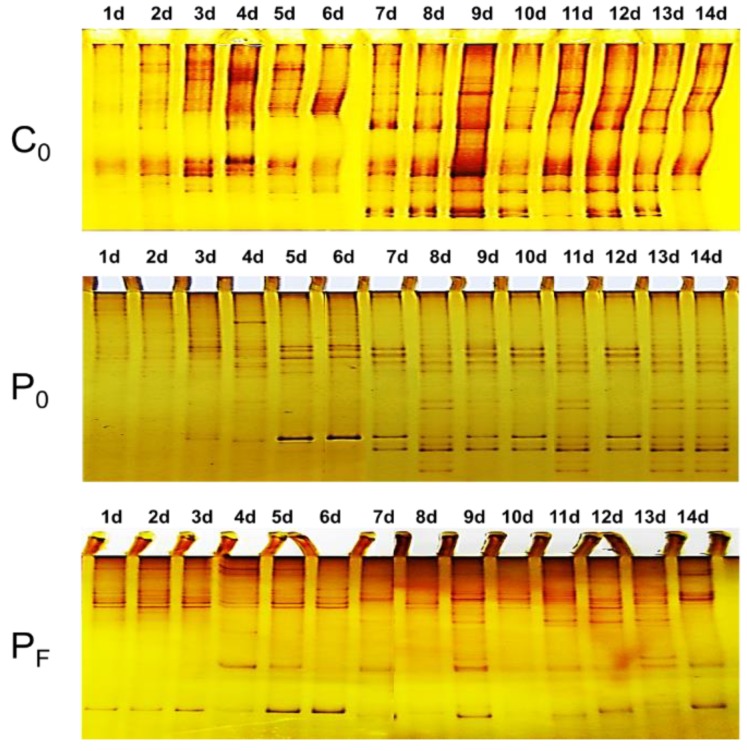
The Single-stranded Conformation Polymorphism (SSCP) patterns of the early biofilm-forming eukaryotic microbial communities developed on the C_0_, P_0_ and P_F_ surfaces.

Figure 3 showed the differential SSCP fingerprints of the pioneer biofilm-forming eukaryotes on the C_0_, P_0_ and P_F_ surfaces during the two-week submergence. As observed from the eukaryotic SSCP fingerprints, the pioneer eukaryotes gradually increased and evolved similar successional patterns on the same type of coating surfaces as a function of time. However, significant differences in the eukaryotic SSCP profiles can be observed and screened among different protective coatings. This suggested the C_0_, P_0_ and P_F_ surfaces have showed differential perturbation effects on the pioneer eukaryotic microbial colonization.

#### 3.3.2. The Eukaryotic Diversity Indices

**Figure 4:**
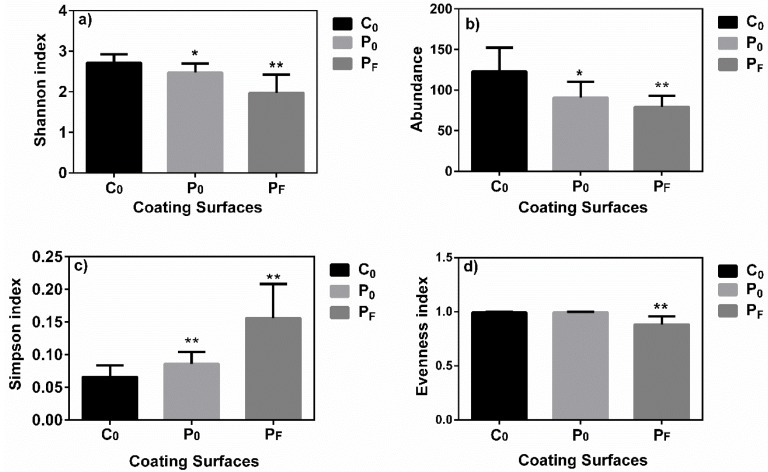
Comparison of the diversity indices of pioneer eukaryotic microbial communities colonized on the C_0_, P_0_ and P_F_ surfaces, including (**a**) Shannon diversity index, (**b**) Abundance, (**c**) Simpson index and (**d**) Evenness index. Error bars represent the standard deviation (SD) of the mean. One asterisk (*) represents significant difference (P < 0.05), whereas two asterisks (**) represent extremely significant difference (P < 0.01).

According to the eukaryotic SSCP profiles, the ecological indices were calculated as presented in Figure 4, which were applied as estimators for further evaluation of the dynamic variations within different pioneer eukaryotic biofilm communities among different protective coating surfaces. In Figure 4a,b, it was clear that the value of the Shannon diversity index and Abundance for the early eukaryotic microbial communities colonized on the C_0_ surfaces (2.71 ± 0.21 and 123 ± 30) were significantly higher than that on the P_0_ surfaces (2.48 ± 0.22 and 91 ± 19) (P < 0.05, ANOVA) and P_F_ surfaces (1.97 ± 0.45 and 79 ± 14), significantly on the P_F_ surfaces (P < 0.01, ANOVA). These combined results indicated that the early eukaryotic microbial diversity and abundance of communities on the PDMS-based coating surfaces (i.e., coating P_0_ and P_F_) were dramatically lower than that on the C_0_ surface during the two-week submergence. This indicated that the C_0_, P_0_ and P_F_ surfaces have demonstrated differential modulating effects on the initial settlement and attachment of the pioneer eukaryotic microorganisms, which may be contributable to accounting for their differential AF performances in the field exposure assays. This remarkably reduced diversity and abundance level suggested that the initial colonization dynamics of early eukaryotic biofilm-forming microbes was greatly perturbed by the PDMS-based coatings, particularly by the PF surfaces. However, this perturbation effect remained relatively weak for the P_0_ and C_0_ surfaces, particularly for the C_0_ surfaces. 

In Figure 4c,d, it is obvious that the value of the Simpson index of the early-adherent eukaryotic microbial communities was found to be greatly enhanced on the P_0_ surfaces (0.156 ± 0.052) and P_F_ surfaces (0.086 ± 0.019), as compared to the C_0_ surface (0.066± 0.018) (P < 0.01, ANOVA). However, the value of the Evenness index of the early eukaryotic communities was significantly reduced on the PF surfaces (0.884 ± 0.074), compared with that on C_0_ surface (0.992 ± 0.007) and the P_0_ surfaces (0.995 ± 0.004) (P < 0.01, ANOVA). These combined results suggested that the distribution patterns and successional dynamics may have been greatly influenced by these protective coatings with surface properties. The dominant eukaryotic microbes in the natural biofilms were found to be greatly increased on the P_0_ and P_F_ surfaces, particularly on the PF surfaces, compared with that of C_0_ surface. The dramatic increase of the dominant eukaryotic microbes suggested that the early eukaryotic microbial communities on P_F_ surfaces were unevenly-distributed, which may have undergone drastic succession process during the two-week submergence, as well-supported by the Evenness data. In contrast, the early eukaryotic microbial communities on C_0_ and P_0_ surfaces were relatively evenly-distributed and therefore possess stable community structures, which were less prone to undergone drastic successional process. Thus, these results also revealed that the cMWCNT nanoparticle incorporation would be contributable to the enhanced perturbation and modulating effect against the initial colonization of pioneer biofilm-forming eukaryotic microbes, as compared to coating C_0_ and the pristine PDMS. 

### 3.4. Clustering Analysis 

**Figure 5:**
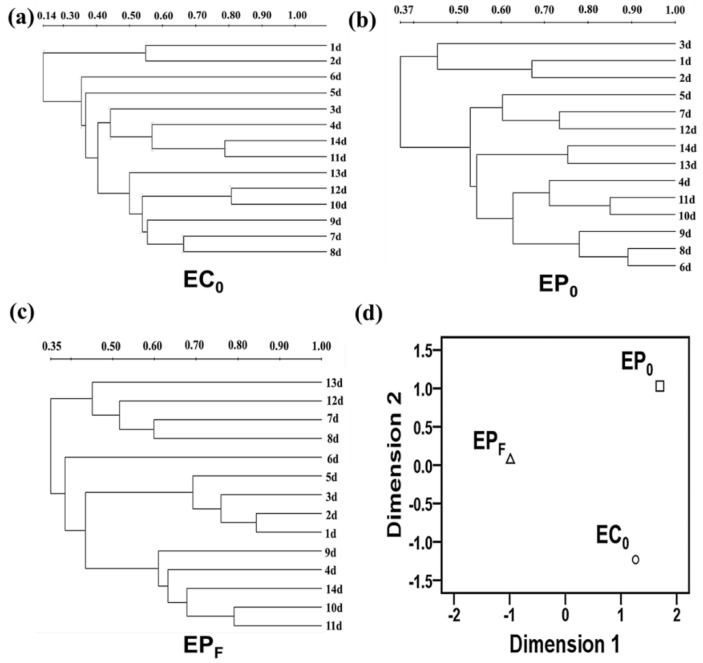
Clustering pattern of pioneer biofilm-forming eukaryotic communities formed on the C0, P_0_ and P_F_ surfaces. (**a–c**) the clustering patterns of pioneer biofilm-forming the eukaryotic communities adhering to the C_0_, P_0_ and P_F_ surfaces (EC_0_, EP_0_ and EP_F_) based on the Unweighted Pair-Group Method with Arithmetic means (UPGMA) method. (**d**) the multidimensional scale (MDS) analysis of the clustering patterns of the early pioneer eukaryotic communities (EC_0_, EP_0_ and EP_F_) on the C_0_, P_0_ and P_F_ surfaces.

Figure 5 showed different clustering patterns of the early-adherent eukaryotic communities on the C_0_, P_0_ and P_F_ surfaces constructed using the UPGMA method (i.e., EC_0_, EP_0_ and EP_F_, see Figure 5a–c) and MDS method (see Figure 5d), respectively. In Figure 5a (EC_0_), it can be seen that most the pioneer eukaryotic biofilm samples, namely samples taken from day 3 to day 14, were liable to cluster into one group, while only two samples taken from day 1 and day 2 tended to cluster into the other group. In Figure 5b (EP_0_), the majority of the biofilm samples taken from day 4 to day 14 were liable to cluster into one group, while only a few samples taken from day 1 to day 3 clustered into the other. In Figure 5c (EP_F_), it is clear that samples taken from day 4, day 6, day 7 and 13, tended to group together, while the other biofilm samples tended to clustered into the other. This differential clustering patterns indicated that there were clear differences within the early biofilm-forming eukaryotic communities adhering to the C_0_, P_0_ and P_F_ surfaces, owing to the differential perturbation effects exerted by different protective coatings. This result was also well supported by the MDS analysis in Figure 5d.

Therefore, these combined results suggested that differences within the surface wettability and morphological characteristics may be primarily responsible for the differential AF performance in the field, thus exerting differential and prominent modulating impacts on the initial colonization and successional dynamics of the pioneer biofilm-forming eukaryotic microbes. It is noteworthy that the complex interactions between the pioneer prokaryotic microbes that colonize these substrata with the early-adherent eukaryotes would also contribute to the differential AF efficacy, which will be reported in a later paper.

## 4. Conclusions

The present study demonstrated that the type of protective coatings and the insertion of CNTs into the plain PDMS would significantly impact the initial colonization and growth of the pioneer biofilm-forming eukaryotic microbes. Polymer–cMWCNT composite materials (P_F_) were proved to be effective in preventing biofouling at different exposure stages, compared with the chlorinated rubber-based coating (C_0_) and the pristine PDMS (P_0_), which seem to be promising materials for future biofilm controls in anti-biofouling applications. Furthermore, the result revealed that the differences within surface properties of these protective coatings may be responsible for the differential perturbation effects and AF efficacy on the early colonization of the early biofilm-forming eukaryotic microorganisms.

## Supplementary Materials

The following are available online at http://www.mdpi.com/2073-4360/11/1/161/s1, Figure S1: Layout of biofilm samples from different protective coatings.
