# Supplementary figures and images for: Differential Colonization Dynamics of Marine Biofilm-Forming Eukaryotic Microbes on Different Protective Coating Materials
Source: Polymers (Basel). 2019 Jan 17;11(1):161. doi: 10.3390/polym11010161 (PMC6401773; doi:10.3390/polym11010161)

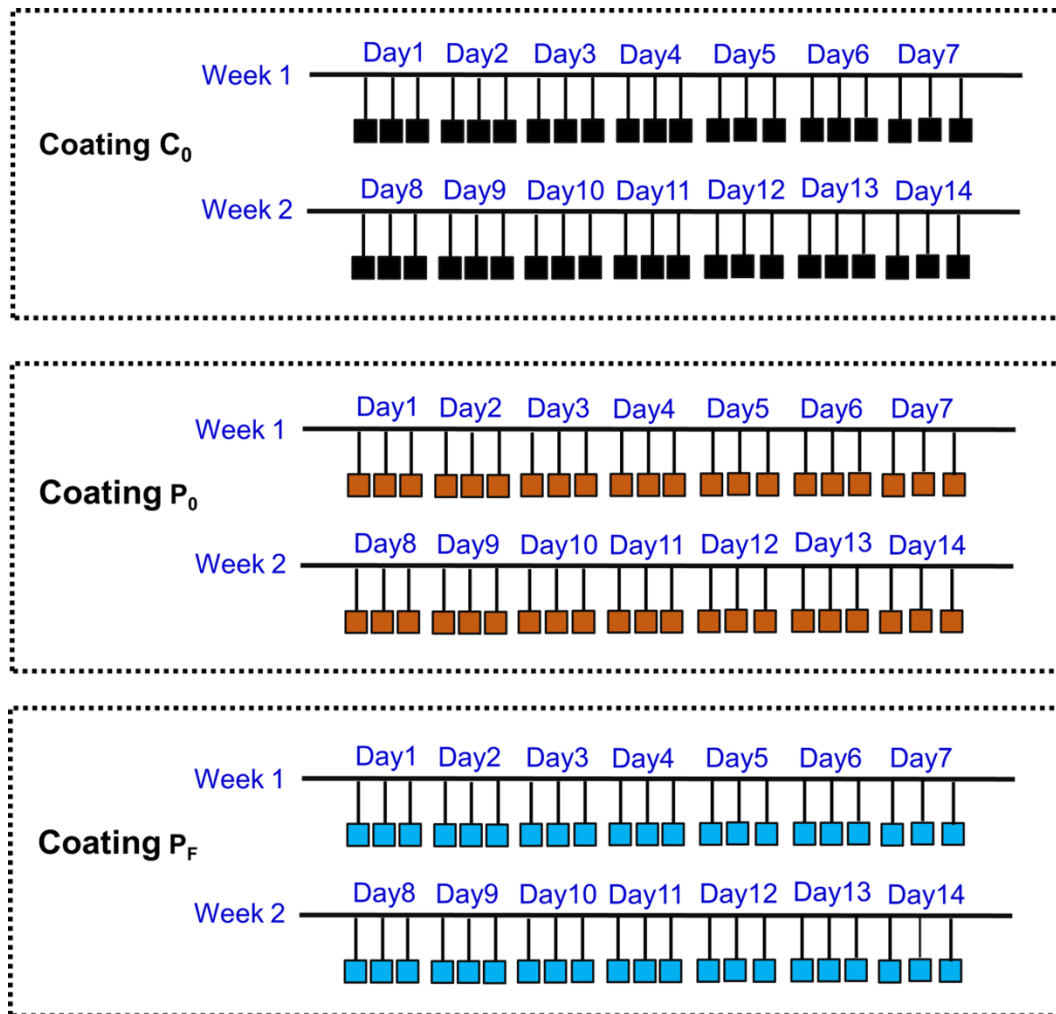

**Figure S1.** Layout of biofilm samples from different protective coatings

Supplement: Supplementary file 1 [file polymers-11-00161-s001.pdf]
